# FcγRI FG-loop functions as a pH sensitive switch for IgG binding and release

**DOI:** 10.3389/fimmu.2023.1100499

**Published:** 2023-02-06

**Authors:** Jinghua Lu, Matthew Spencer, Zhongcheng Zou, Maria Traver, Joseph Brzostowski, Peter D. Sun

**Affiliations:** ^1^ Structural Immunology Section, Lab of Immunogenetics, National Institute of Allergy and Infectious Diseases, National Institutes of Health, Rockville, MD, United States; ^2^ Lymphocyte Activation Section, Lab of Immunogenetics, National Institute of Allergy and Infectious Diseases, National Institutes of Health, Rockville, MD, United States

**Keywords:** human FcγRI, antibody recognition, receptor FG-loop, Fc-glycan recognition, pH sensitive binding, live cell tracking of FcγRI-antibody endocytosis

## Abstract

Understanding the molecular mechanism underlying the hierarchic binding between FcγRs and IgG antibodies is critical for therapeutic antibody engineering and FcγR functions. The recent determination of crystal structures of FcγRI-Fc complexes, however, resulted in two controversial mechanisms for the high affinity receptor binding to IgG. Here, we describe high resolution structures of a bovine FG-loop variant of FcγRI in complex with the Fc fragment of IgG_1_ crystallized in three different conditions at neutral pH, confirming the characteristic FG loop-Fc interaction is critical to the high affinity immunoglobulin binding. We showed that the FcγRI D2-domain FG-loop functioned as a pH-sensing switch for IgG binding. Further live cell imaging of FcγRI-mediated internalization of immune complexes showed a pH sensitive temporal-spatial antibody-antigen uptake and release. Taken together, we demonstrate that the structures of FcγRI-Fc crystallized at neutral and acidic pH, respectively, represent the high and low affinity binding states of the receptor for IgG uptake and release. These results support a role for FcγRI in antigen delivery, highlight the importance of Fc glycan in antibody binding to the high affinity receptor and provide new insights to future antibody engineering.

## Introduction

Therapeutic IgG antibodies have been extensively developed during the past decades to treat various diseases, such as cancer, infectious diseases, allergies, and Alzheimer’s diseases. Numerous investigations have been dedicated to reveal the mechanisms whereby therapeutic antibodies exert their biological effects. Among them, membrane bound Fcγ receptors (FcγRs) are the key effectors and regulators of IgG mediated immune response due to the different characteristics of heterogeneous FcγRs. According to the binding affinities, FcγRs can be categorized as high or low affinity receptors and even a given FcγR could bind to different IgG subclasses with different affinities. In addition, the glycosylation modifications of both FcγRs and IgGs modulate the binding affinities of their interactions. Upon binding of IgG/antigen complexes, FcγRs can initiate totally opposite cellular responses, which depend on the activating or inhibitory immunoreceptor tyrosine signaling motif that they bear or associate within the cytoplasm. Finally, the biological effect mediated by FcγR-IgG binding is a temporal-spatial cellular process spanning from the cell surface to the intracellular endosome/phagosome. Owing to the rapid progress of bioengineering technology, therapeutic antibodies are also developed to have tailored binding properties and improved efficacy and half-lives. Altogether, the basis of therapeutic IgG antibody development and engineering relies heavily on the understanding of the molecular binding mechanisms of FcγR-IgG interactions.

Although earlier structural studies described the binding mode between low affinity receptor and IgG, the structural mechanism underlying the high affinity FcγRI-IgG binding remain unresolved. Among the known FcγRs, FcγRI is the only receptor with high affinity to bind selective subclasses of IgG in monomeric form (1–4). The function of FcγRI remains a conundrum as the receptor is thought to be saturated by high concentrations of circulating IgG. Yet, evidence supports roles for FcγRI in antibody-mediated cellular responses (2, 3, 5). Unlike the low affinity FcγRII and FcγRIII, FcγRI is not only broadly expressed on myeloid cells, but also an inflammatory receptor, whose expression is upregulated by IFN-γ (6–8). In addition, deficiency of FcγRI affected antibody-dependent responses (9). On the other hand, FcγRI binding of pathogenic autoantibodies was shown to exacerbate some autoimmune conditions, such as rheumatoid arthritis (10). Owing to its high affinity IgG binding, FcγRI is also a target of antibody engineering to modulate its binding affinity to therapeutic antibodies (11).

The structures of the extracellular domain of the high affinity IgG receptor FcγRI in complex with IgG_1_-Fc have recently been published (12–14). These structures showed that the high affinity FcγRI docked on IgG in a similar mode to those of the low affinity FcγR receptors. Nevertheless, structural discrepancies led two contradicting structural mechanisms for the high affinity binding between FcγRI and IgG (12–15). Further, assumptions were made to attribute the structural discrepancies to the crystallographic resolutions with the implication that the binding mechanism deduced from the lower resolution complex structure was incorrect (13–15). Given its unique high affinity IgG binding as well as its potential role in both antibody-mediated cellular humoral response and antibody engineering for immunotherapy, we attempted to resolve the conflicting high affinity binding mechanism proposed for FcγRI. To address the crystallographic resolution-related discrepancy in proposed binding mechanisms, we determined a bovine FG-loop variant of FcγRI in complex IgG_1_-Fc to 2.3-2.5 Å resolutions, demonstrating that the crystallographic resolution was not responsible for the structural discrepancies. This was further supported by mechanism-specific mutational analyses of the receptor. In addition to various crystallographic resolutions, the crystallization conditions among of the published FcγRI-Fc complexes also differed. To probe if crystallization conditions influenced the receptor-ligand binding, we further carried out pH-dependent solution binding experiments between FcγRI and IgG, and tracked an antibody-antigen complex internalization by the receptor during phagocytosis using confocal and live cell fluorescent imaging experiments. Taken together, these experiments reconciled the structural discrepancies in published FcγRI-Fc structures and demonstrated the high and low affinity conformations in FcγRI-IgG binding, were regulated by the receptor and Fc-glycan interactions in response to intracellular pH environment. Such cellular temporal-spatial binding and release of IgG by FcγRI favors the involvement of the receptor in antigen presentation of small soluble immune complexes.

## Results

### Positively charged D2 domain FG-loop is unique to FcγRI but not FcγRII or FcγRIII

**Figure 1:**
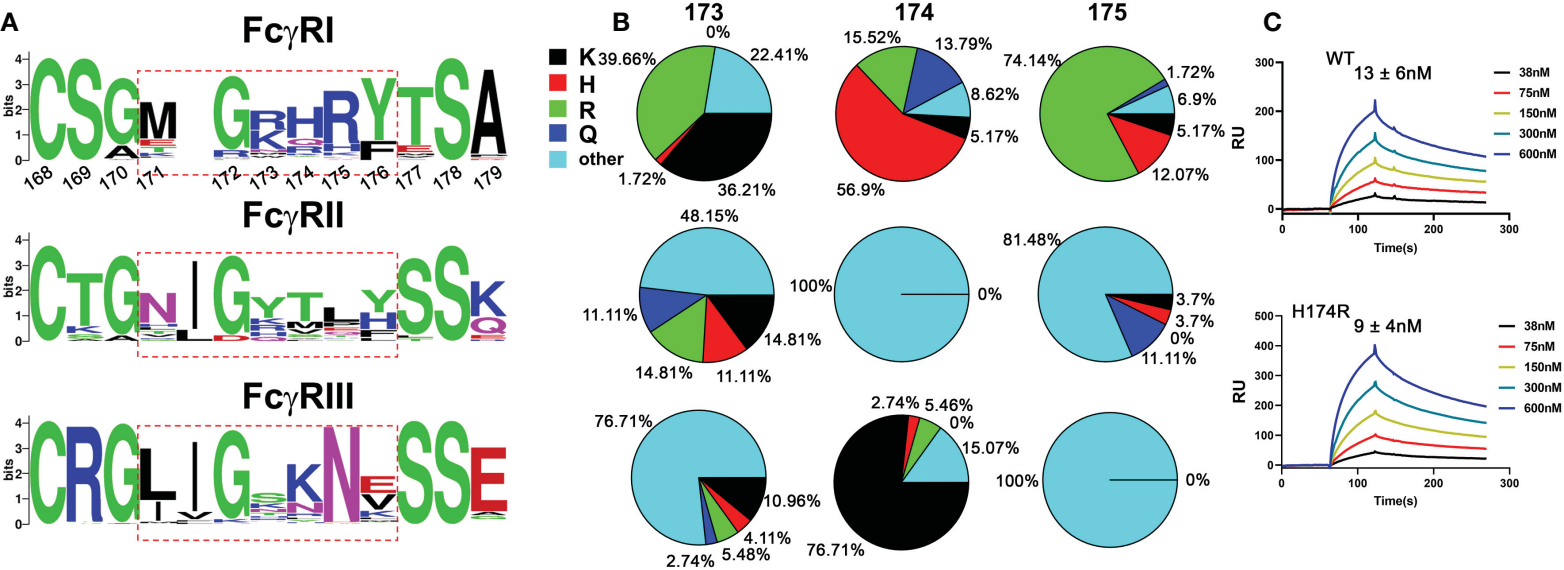
Sequence analysis of FG loop residues in FcγRs D2 domain. **(A)** Logo display of FG loop residues among all FcγRs. **(B)** Usage of positively charged residues K,H,R, and other residues on FG loop. **(C)** Binding affinities of human and bovine H174R variant FcγRI to human IgG_1_.

All known Fcγ receptors recognize the lower hinge region of IgG with their receptor D2 domain (15, 16). The conserved receptor-Fc docking mode results in primarily two similar contact regions between FcγR and the lower hinge regions of two IgG-Fc chains. In the case of FcγRI, the lower hinge of IgG-Fc A chain, referred to as hinge A region, forms primarily hydrogen bond interactions with residues on strand C and C’ of FcγRI, while the lower hinge of IgG-Fc B chain, referred to as hinge B region, forms mainly hydrophobic interactions that include the “WPW” sandwich between Pro 329 of Fc and Trp 104, Trp 127 of the receptor. Many of the receptor residues contacting hinge A and B of Fc are conserved between the high affinity FcγRI and the low affinity FcγRII, FcγRIII, making it difficult to deduce the critical contacts responsible for the high affinity binding of FcγRI. In addition to the conserved receptor contacts to hinge A and B of Fc, the FcγRI D2 domain FG-loop, residues 171-176, was observed to form additional contacts with Fc in the FcγRI-Fc complex structure by Lu et al (12). These FG-loop-mediated contacts, however, were not observed in the other two complexes (13, 14). Unique to the high affinity FcγRI is the presence of three consecutive positively charged residues 173-175 in its FG-loop, which are not observed in the low affinity FcγRII and FcγRIII receptors (**Figure 1A**) (12). The appearance of the charged residues is quite conserved in all mammalian FcγRI with over 75% of amino acids being positively charged at each of the position 173, 174 and 175 (**Figure 1B**). In comparison, FcγRII and FcγRIII carry no more than one positively charged residue in their corresponding sequences (**Figure 1B**). Further, the charged amino acids in FcγRI FG-loop also exhibit residue preference. Arg and Lys occur equally (~36%-40% each) at position 173 with His counting for less than 2% of the sequences. In contrast, His is observed in over 50% of the sequences at position 174, followed by Arg (~15%) with Lys counting for ~5% of the sequences. Position 175 is dominated with Arg (~75% of the sequences). These results suggest the selective usage of positively charged amino acids in the high affinity receptor FG-loop is not random, rather a distinct feature with potential functional advantage. Indeed, the replacement of FG loop of FcγRIII with FcγRI FG loop dramatically increased the binding affinity of FcγRIII to IgG_1_ (17). In addition, the mutations of these positively charged residues on FcγRI FG loop to Ala or Glu significantly affected the high affinity interaction between FcγRI and IgG (12).

### Structure of FcγRI H174R variant in complex with Fc

**Figure 2:**
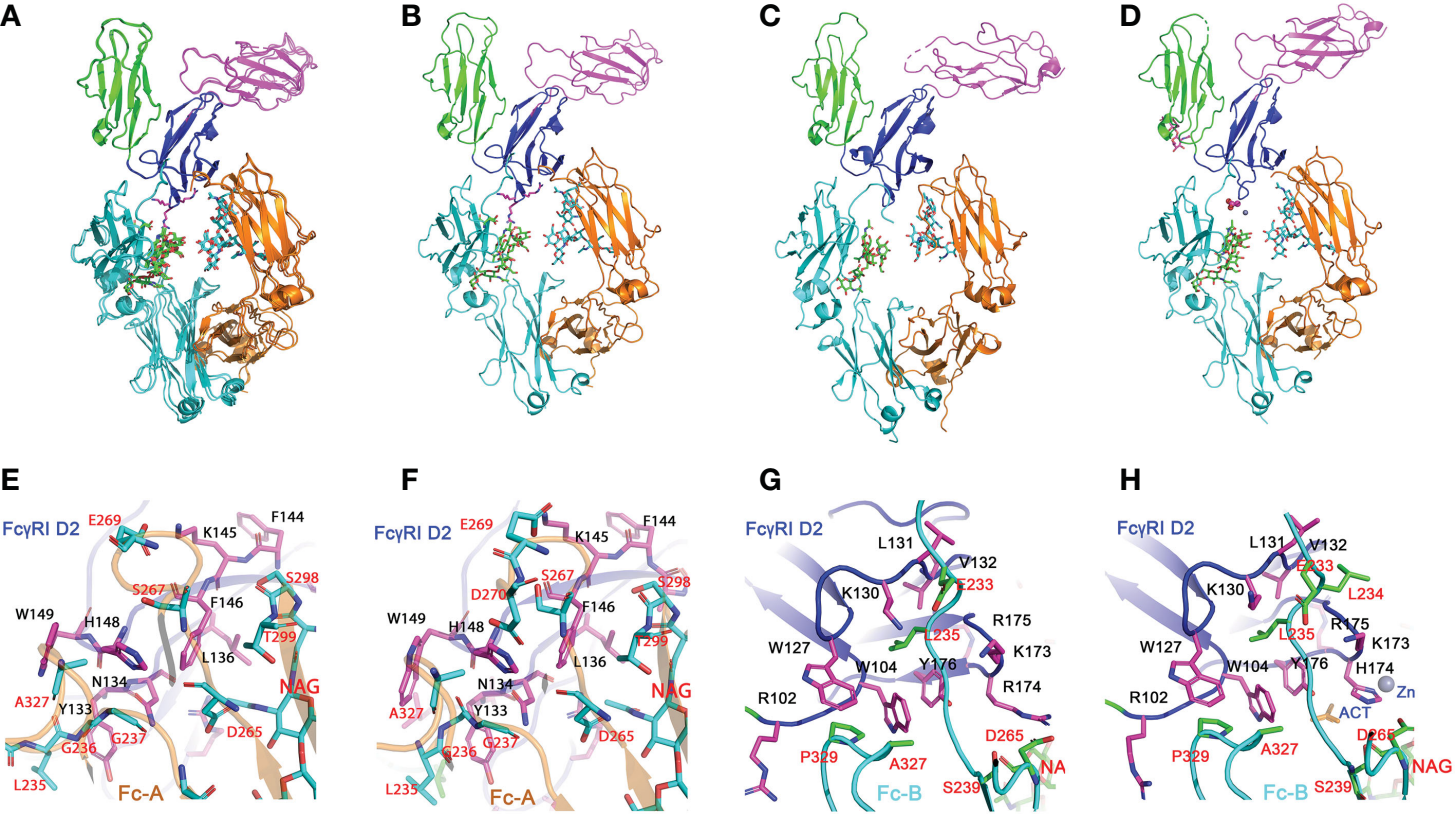
Structures of H174R FcγRI in complex with IgG_1_ Fc. **(A)** Structure superposition of three structures of FcγRI H174R variant in complex with Fc (PDB code: 8DIR, 8DIN and 8DJ7). All structural alignments were based on D2 domain of FcγRI. D1, D2 and D3 domain of FcγRI were highlighted as cartoons in green, blue, and magenta, respectively. The Fc chain **(A, B)** were colored in orange and cyan, respectively, with glycosylation attached to Asn 297 shown in stick model. **(B, D)** Structures of individual FcγRI-Fc complexes, 8DIR **(B)**, 4X4M **(C)** and 4W4O **(D)**. The acetate and Zn atom bound to H174 are shown in spheres. E-F) Interface contact region between FcγRI D2 domain and Fc-A chain as observed in 8DIR **(E)** and 4W4O **(F)**. **(G, H)** Interface between H174R FcγRI D2 domain and Fc-B chains observed in 8DIR **(G)** and 4W4O **(H)**. FcγRI residues were shown as magenta sticks and labelled in black. Residues from Fc chains were shown as cyan or green sticks and labelled in red.

The recent publication of three FcγRI-Fc complex structures yielded two conflicting mechanisms regarding to the main determinant of the receptor’s high affinity IgG binding (12, 13). While Lu et al. proposed the high affinity IgG binding resulted from unique interactions between FcγRI D2 domain FG-loop and Fc, Kiyoshi et al. emphasized a hydrophobic packing between the receptor and Leu 235 of Fc as the high affinity IgG binding mechanism. To further resolve the conflicting mechanism, we examined sequence variants of the receptor D2 domain FG-loop assuming the high affinity IgG binding is retained by FcγRI from other species. Since Arg is the second abundant amino acid at the FG-loop position 174 and is observed in bovine and ovine FcγRI sequences, we generated a H174R mutant of human FcγRI. Indeed, the H174R variant of FcγRI bound to all IgG isotypes with similar affinity as the wildtype receptor (**Table 1** and **Figure 1C**). We subsequently crystallized the bovine FG-loop variant of FcγRI in complex with human IgG_1_-Fc in three different salt conditions at neutral pH (**Table S1**). All three crystal forms belonged to the same crystallographic lattice with similar unit cell dimensions and their structures were determined by molecular replacement to high resolutions between 2.3 and 2.5Å, respectively (**Table S1**). The three complex structures were nearly identical with root-mean-square (rms) deviations of 0.2-0.25 Å among them (**Figure 2A**).

**Table 1:** Dissociation constants for FcγR binding to IgG.

FcγRI	Affinity (K_D_, nM)			
	IgG_1_	IgG_2_	IgG_3_	IgG_4_
WT	13.0±6	41.0±29.3	28.6±19.9	65.7±53.0
H174R	9.0±4	32.8±12.5	24.2±18.5	108.8±54.7
H174E	250.6±115			
K173A/H174A/R175A (AAA)	180.0±42			
K173E/H174E/R175E (EEE)	418 ± 198			
V132L/Y176V	25.8±28.2			
FcγRIII	IgG_1_(K_D_, µM)			
WT	3.4±9			
L118V	14.7±1.9			
V163Y	4.4±2.1			
L118V/V163Y	4.8±0.4			
	pH-dependent binding to anti-BSA (K_D_, nM)			
	7.5	4.6	4.0	
FcγRI	13.5±3.8	193±176	ND	
BSA	7.8±1.8	10.1±14.5	1.8±1.1	

**Figure 3:**
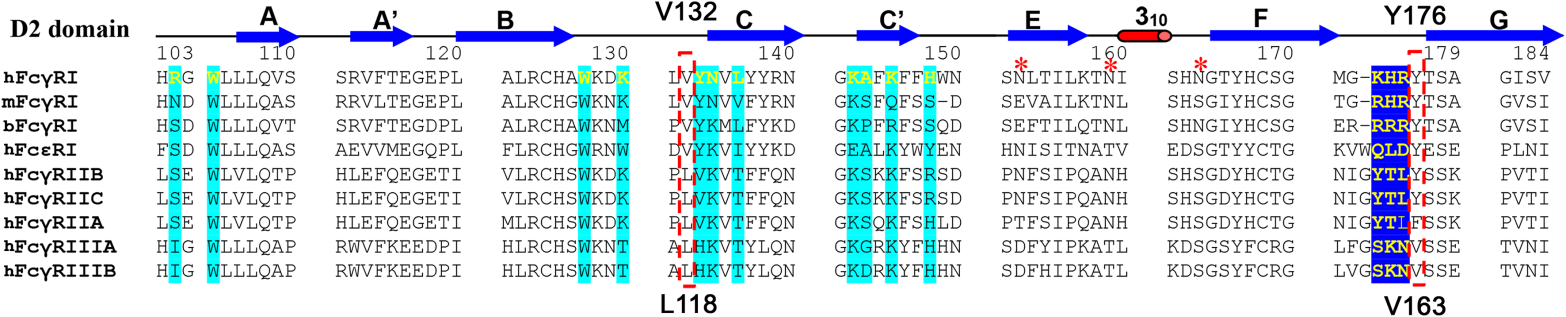
Sequence alignment of FcγR D2 domains. The interface residues in FcγRI-Fc complex are shaded in cyan or blue. V132 and Y176 of FcγRI are highlighted in the red box. They correspond to L118 and V163 in FcγRIII.

The overall docking of FcγRI H174R variant receptor on Fc closely resembles those previously observed (12–14) (**Figures 2B–D**) in which the D2 domain of FcγRI is wedged into the horseshoe opening of Fc to induce a conformational change in Fc from a symmetric to asymmetric dimer. The docking of FcγRI on Fc is in fact the same as those observed in all low affinity FcγRII- and FcγRIII-Fc complex structures (18–21), further highlighting the common binding mode between Fc and FcγRs and their conserved interactions. In brief, the A chain of Fc interacts with the C-strand (Tyr 133 to Leu 136), C’-strand (Lys 142 to His 148) and C’E loop (His 148 to Trp 149) of FcγRI D2-domain. The receptor forms primarily polar interactions with Fc residues at lower hinge A. These include 4 hydrogen bonds involving Asn 134, Lys 142, and His 148 of FcγRI and one salt bridge between Lys 145 of FcγRI and Glu 269 of Fc-A (**Table S2** and **Figures 2E, F**). The B chain of Fc interacts with the receptor D1-D2 interdomain hinge region (Arg 102 to Trp 104) and BC loop (Trp 127 to Tyr 133) of the D2 domain (**Figures 2G, H**). Trp 104 and Trp 127 of FcγRI form a tryptophan sandwich with Pro 329 of Fc at the lower hinge-B contact region. This WPW sandwich is conserved in both high and low affinity FcγRs (**Figure 3**). However, the overall interface area between the FcγRI receptor and Fc is ~1270Å^2^, approximately 400-500 Å^2^ more than those of low affinity FcγRIII-Fc complexes. Most of the interface difference is the result of additional contacts between FcγRI D2-domain FG loop and Fc.

### Contacts between FcγRI D2 domain FG-loop and Fc

**Figure 4:**
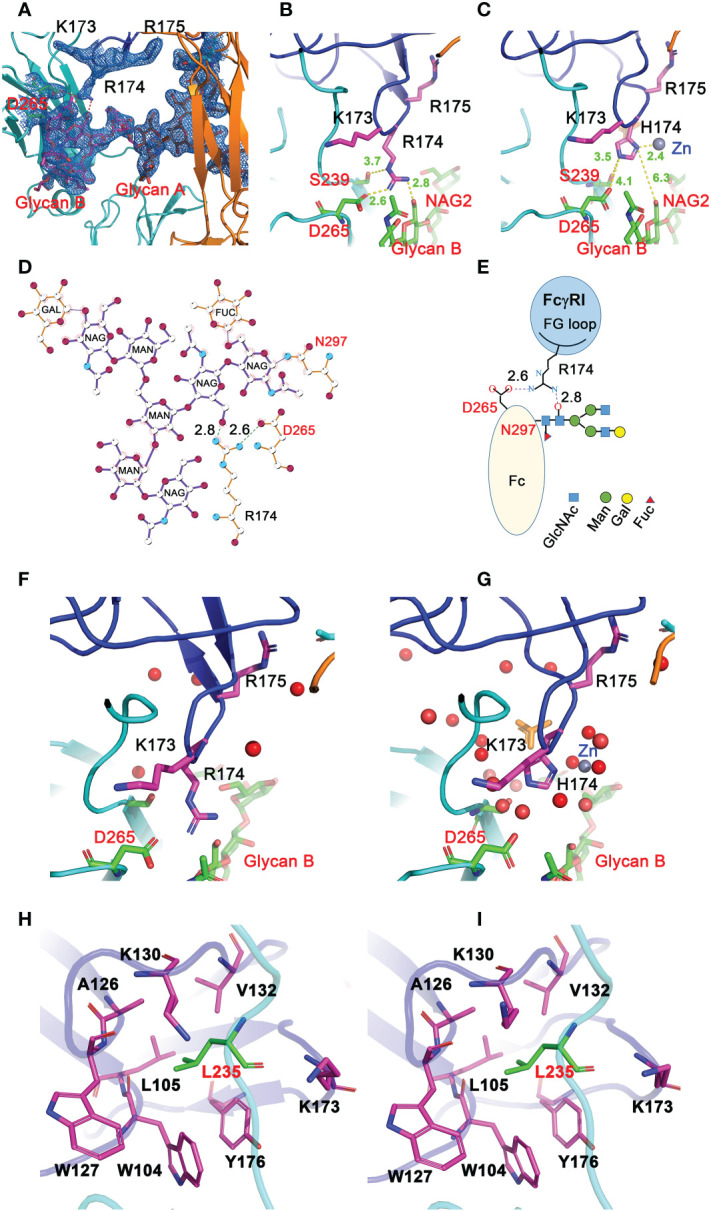
Interactions between FG loop and Fc. **(A)** (2Fo-Fc) electron density map contoured at 1σ showing R174 of FcγRI forming salt-bridge and hydrogen bond contacts with D265 and glycan, respectively, on Fc. **(B)** Stick model depicting R174 contacting D265 and glycan on Fc in the H174R variant FcγRI-Fc complex. **(C)** H174 in 4W4O is situated too far from D265 and glycan but instead is coordinated by a Zn ion. **(D, E)** Ligplot and cartoon drawing to illustrate the interactions from R174 of the receptor to D265 and glycans on Fc-B chain. **(F, G)** interactions between R174 and Fc observed in 8DIR **(F)** are replaced by water molecules in 4W4O **(G)**. **(H, I)** Contacts of Fc-B chain L235 with the receptor hydrophobic pocket as observed in 8DIR **(H)** and 4W4O **(I)**.

All three structures crystallized at neutral pH with different salts contain well-defined electron densities at the interface between the receptor FG-loop and Fc (**Figure 4A** and **Figure S1**). Arg 174 on the receptor FG-loop forms two contacts with the B-chain of Fc, including a salt bridge to Asp 265 and a hydrogen-bond to the hydroxyl group of the second N-acetylglucosamine (GlcNAc labeled as residue NAG502) on the glycan associated with Asn 297 on the Fc (**Figures 4B, D, E**). As the N-linked glycan on Asn 297 is conserved in all isotypes of IgG and the second GlcNAc is conserved in all N-linked glycans, the result shows a conserved FcγRI-glycan interaction with all IgG isotypes. It is worth noting that a recent study of human FcγRI binding to IgG_1_ by hydrogen-deuterium exchange mass spectrometry also supports the direct FG-loop and glycan interaction (22). These contacts between the FG-loop and Fc were not observed in complex structures by Kiyoshi et al. (PDB entry 4W4O) and Oganesyan et al. (PDB entry 4ZNE) (13, 14). In both structures, the receptor FG-loop is retracted ~2 Å away from the Fc. The FG-loop residue 174 was instead coordinated by a Zn ion and an acetate from the crystallization solution (**Figure 4C** and **Table S2**). The lack of direct contacts in 4W4O between FcγRI FG-loop and Fc is also evident from the presence of numerous water molecules at the interface. While there are 5 water molecules observed between the receptor FG-loop and Fc in the current H174R complex, there are additional 11 water molecules in the same region of 4W4O (**Figures 4F, G**). These additional water molecules form an extensive hydrogen-bond network in 4W4O, separating the receptor FG-loop from direct contacting with Fc. These results showed that the largest differences between the two complex structures (H174R-Fc and 4W4O) were the receptor FG-loop mediated Fc contacts. As the structure 4ZNE by Oganesyan et al. is essentially identical to that of Kiyoshi et al., all structural comparisons here refer to the structure by Kiyoshi et al.

### FcγRI FG-loop residues determine the high affinity IgG binding

**Figure 5:**
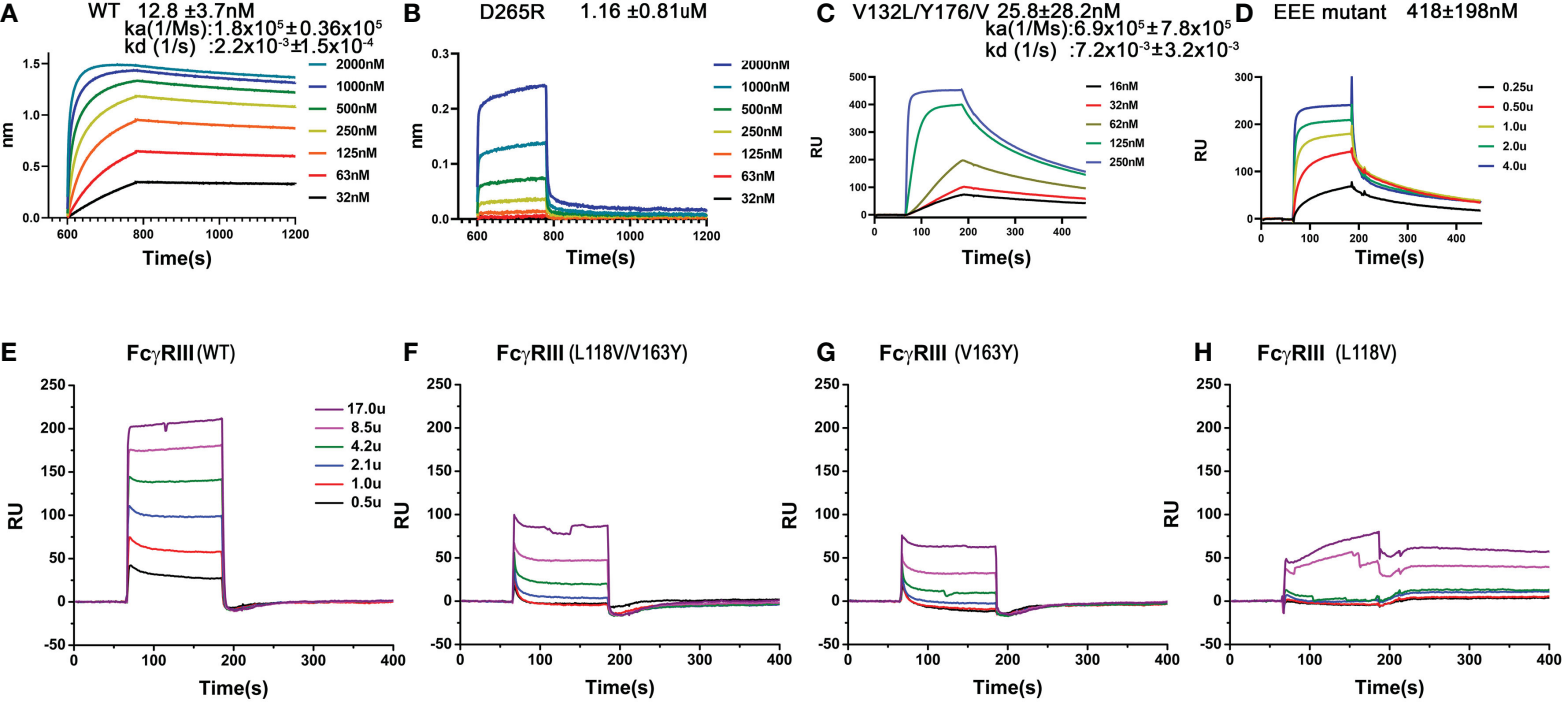
Binding of wildtype or mutant recombinant FcγRI and FcγRIII to IgG_1_. **(A, B)** Biolayer interferometry (BLI) measurements of FcγRI binding to the wildtype and D265R mutant of VRC01. **(C, D)** SPR measurements of FcγRI V132L/Y176V **(C)** and ^173^KHR^175^ to ^173^EEE^175^ **(D)** mutants binding to IgG_1_. **(E–H)** SPR measurements of FcγRIII WT and mutants binding to IgG_1_.

Furthermore, these FG-loop mediated Fc contacts are also unique to the high affinity receptor FcγRI and are not present in the low affinity Fc-receptors. In addition, the FG-loop of human FcγRI D2 domain is one amino acid shorter than those of the low affinity receptors (12). The replacement of the FG-loop in FcγRIII with that of FcγRI resulted in > 10-fold increase in IgG_1_ binding affinity (17). When a valine residue was inserted into the FcγRI FG-loop, it resulted in an 8-fold reduction in IgG_1_ binding affinity (17). When residues 173-175 (KHR) of the FcγRI FG-loop were replaced with alanine, the KHR to AAA mutation resulted in a ~15-fold affinity reduction to 180nM in the receptor binding to IgG_1_ (**Table 1**). The contribution of FcγRI FG-loop charge to the receptor binding to IgG was evident when histidine 174 was replaced with Glu, resulting in a ~20-fold loss in IgG_1_ binding (**Table 1**). Replacing all three positively charged KHR motif in the FG-loop with Glu (EEE mutant) resulted in ~40-fold loss in the receptor binding to IgG (**Figure 5D**). Part of the additional affinity loss associated with the negatively charged EEE mutant is likely due to a repulsive interaction with Asp 265, a salt bridge partner of the FG-loop Arg 174. Conversely, when Asp 265 on the heavy chain of a human IgG_1_ isotype anti-gp120 antibody, VRC01, was replaced with Arg, the D265R mutant VRC01 antibody bound to the wildtype FcγRI with 1.16 µM affinity, a ~100-fold loss compared to the 12 nM FcγRI binding affinity of the wildtype VRC01 (**Figures 5A, B**). Thus, either replacing positively charged residues in the FG-loop of the receptor with negatively charged residues or replacing negatively charged Asp 265 of Fc with positively charged Arg dramatically reduced the receptor-ligand binding, supporting that the FcγRI FG-loop determines the high affinity interaction with IgG.

### Mutations of the receptor residues forming the hydrophobic core with lower hinge-B of Fc

As the receptor FG-loop made no direct contacts to Fc in the complex 4W4O, Kiyoshi et al. proposed a receptor-induced conformational change in the lower hinge region of Fc-B as the mechanism for the high affinity FcγRI binding to IgG. This conformational change results in the insertion of Leu 235 into a hydrophobic pocket formed by Trp 104, Leu 105, Lys 130, Val 132, and Tyr 176 of FcγRI (**Figures 4H, I**) (13). Three of the five hydrophobic pocket forming residues, Trp 104, Leu 105 and Lys 130, are conserved between the high affinity FcγRI and the low affinity FcγRIII. Val 132 and Tyr 176 are conserved only in FcγRI sequences. Val 132 is replaced by Leu in all lower affinity FcγRII and FcγRIII sequences (**Figure 3**). Tyr 176 is replaced by either Phe or Val in FcγRIIA and FcγRIII. Both Val 132 and Tyr 176 were proposed to be important for the receptor’s high affinity IgG binding (13). To assess the potential contribution of the hydrophobic pocket to the high affinity IgG binding of FcγRI, we performed mutational analyses to replace Val 132 and Tyr 176 of FcγRI with their corresponding residues in FcγRIII (**Figure 3**), as well as to replace the corresponding Leu 118 and Val 163 in FcγRIII with Val and Tyr, respectively, to construct a high affinity receptor-like hydrophobic core in the low affinity FcγRIII.

The FcγRI V132L/Y176V double mutant only exhibited a ~2-fold reduction in IgG binding affinity of 25.8±28.2nM (**Figure 5C**), less dramatic compared to the 15-30 fold reduction of the FG-loop mutants: H174E, AAA or EEE (**Figure 5D** and **Table 1**). Conversely, when Leu 118 and Val 163 in FcγRIII were mutated to their corresponding FcγRI residues, Val and Tyr, respectively, both the single mutants L118V and V163Y, as well as the double mutant L118V/V163Y resulted in equivalent or reduced IgG_1_ binding affinity compared to that of the wildtype FcγRIII (**Figures 5E–H** and **Table 1**). In contrast, when the FG-loop in FcγRIII was replaced with that of FcγRI, it resulted ~15-fold improvement in IgG binding (17). The failure to gain IgG_1_ binding affinity when the lower hinge-B hydrophobic core lining residues of FcγRIII were mutated to those of FcγRI suggests these lower hinge-B hydrophobic core lining residues are not critical for FcγRI high affinity IgG binding. Scrutinization of the detailed interactions of L235 on Fc B chain with both FcγRI and FcγRIII reveals that the majority of VDW and hydrogen bonding interactions that involve L235 come from main chain atoms, suggesting that the N terminus of the Fc hinge functions as an anchor cable while the Fc adopts the asymmetric conformational changes to dock onto FcγRs. Indeed, effector cell bioassays showed engineered CD20 antibodies with L234A/L235A(LALA) mutations gave equivalent responses *via* FcγRI but lost substantial activities with low affinity receptors FcγRIIa and FcγRIII. One additional mutation P329G in the conserved WPW sandwich (L234A/L235A/P329G) further abolished cellular responses with all the Fcγ receptors, highlighting the contribution of hinge loop and WPW sandwich to the common binding mode between FcγRs and IgG antibodies (23).

### High affinity FcγRI and IgG binding is sensitive to pH

**Figure 6:**
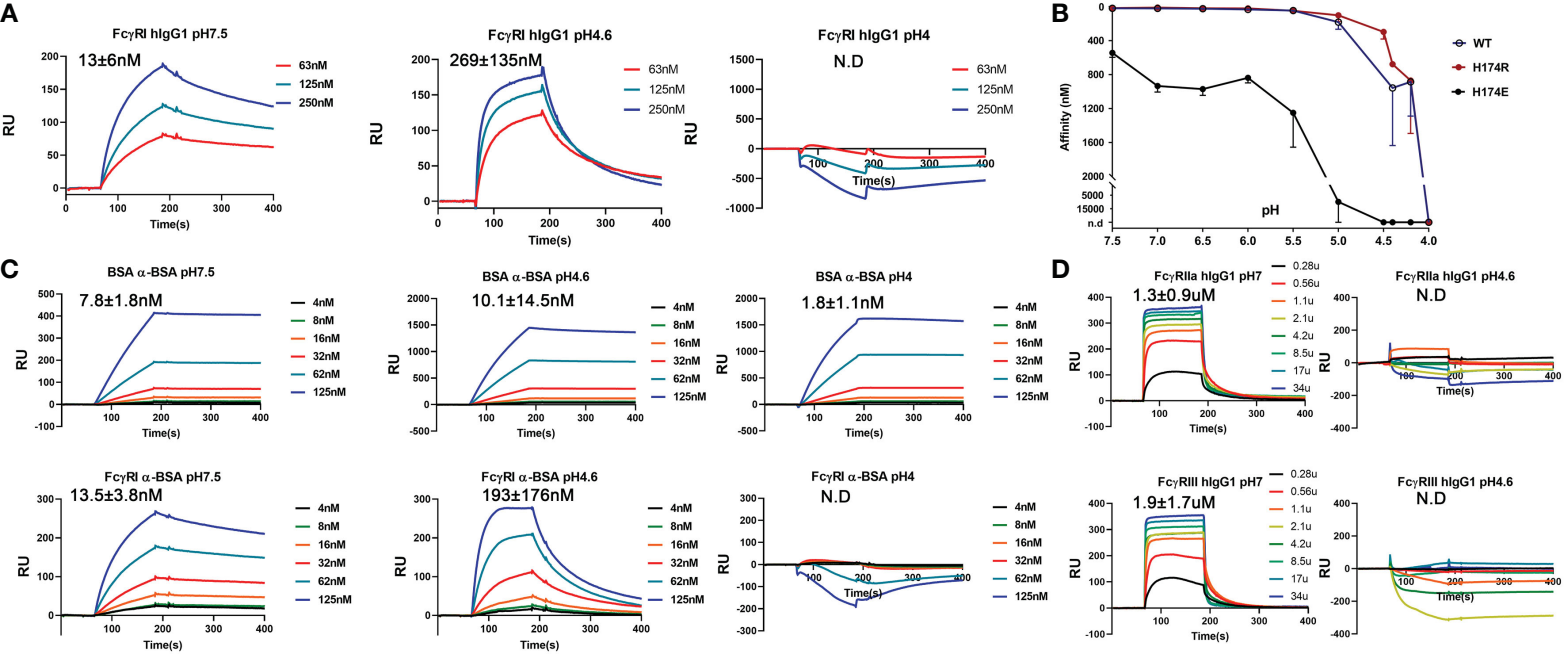
pH sensitive binding between FcγRI and IgG. **(A)** Binding of FcγRI to IgG_1_ at pH 7.5, 4.6 and 4.0. **(B)** pH-titration of the FcγRI wildtype, H174R and H174E mutants binding to IgG_1_. **(C)** Binding of BSA and FcγRI to rabbit polyclonal anti-BSA antibody at pH7.5, 4.6 and 4.0. **(D)** Binding of FcγRII and FcγRIII to IgG_1_ at pH7 and 4.6.

To reconcile the observed FG-loop structural differences in different FcγRI-Fc complexes, we noticed differences in the crystallization conditions of all three published structures. The early FcγRI-Fc complex by Lu et al. (4X4M) was crystallized in 10% PEG 8000, 50mM Li_2_SO_4_ and 10mM HEPES/pH 7.5 while the one by Kiyoshi et al. (4W4O) was crystallized in 0.1M sodium acetate, 0.1M zinc acetate, 4% 1,4-butanediol and 12% PEG 4000 at pH 4.6 (12, 13). The structure obtained by Oganesyan et al. (4ZNE) was crystallized into the same space group as that by Kiyoshi et al. under similar conditions of PEG 3350 and zinc acetate (13, 14). The current bovine FG-loop variant and Fc complex was crystallized in 20% PEG3350 with 0.2 M of different salts at pH 6.6-7.5. Since the FcγRI FG-loop residues in both complexes crystallized from zinc acetate did not contact Fc but instead showed the coordination of the FG-loop His 174 to zinc and acetate groups in Kiyoshi et al. (**Figure 4G**), we reasoned that the presence of acetate competes with the FG-loop salt bridge to Asp 265 of Fc and hydrogen bond to the hydroxyl group on the second N-acetylglucosamine associated with Asn 297. As higher acetate concentration is usually associated with lower pH, we then investigated if FcγRI-IgG interaction was sensitive to pH by measuring the binding affinities of recombinant FcγRI to IgG_1_ between pH 7.5-4 using BIAcore. Both the His and Arg 174 forms of FcγRI bound to IgG_1_ with ~10 nM affinity at neutral pH and remained high affinity binding to IgG above pH 5.5, but exhibited pH dependent affinity reduction below that (**Figures 6A, B** and **Table 1**). At pH 4.6 the receptor exhibited ~269 nM binding affinity for IgG_1_, similar to that of AAA and EEE mutant binding at neutral pH (**Table 1** and **Figure 6A**). No binding to IgG could be detected for FcγRI at pH 4 (**Figures 6A, B**). As a comparison, we measured an antibody-antigen binding using bovine serum albumin (BSA) and anti-BSA rabbit polyclonal antibodies at both neutral and acidic pH conditions. Both BSA and FcγRI bound to anti-BSA with similar high affinities of ~10 nM at neutral pH (**Figure 6C**). While BSA binding to anti-BSA remained high affinity between pH 7.5 and 4, FcγRI binding to anti-BSA showed the characteristic pH dependent affinity reduction (**Figure 6C**), suggesting the pH dependent binding is a unique feature of FcγRI. Since the binding affinity between IgG and FcγRI is significantly lower at low pH, we conclude that the binding mode in the complexes crystallized at low pH represents a low affinity binding state of FcγRI-IgG interaction due to the absence of the FG-loop and Fc interactions. Consistently, the IgG binding affinity of the FG-loop His 174 to Glu mutation (H174E) exhibited an earlier transition between pH 6-5 and was undetectable at pH 4.5 (**Figure 6B**). Similarly, both low affinity FcγRIIa and FcγRIII binding to IgG became undetectable at pH 4.6 (**Figure 6D**). All together, these results showed that FG-loop interaction with Fc determined the high affinity binding of FcγRI to IgG at neutral pH and FG-loop could mediate a pH-dependent dissociation between FcγRI and IgG.

### Tracking FcγRI-mediated release of antibody-antigen complex in cells

**Figure 7:**
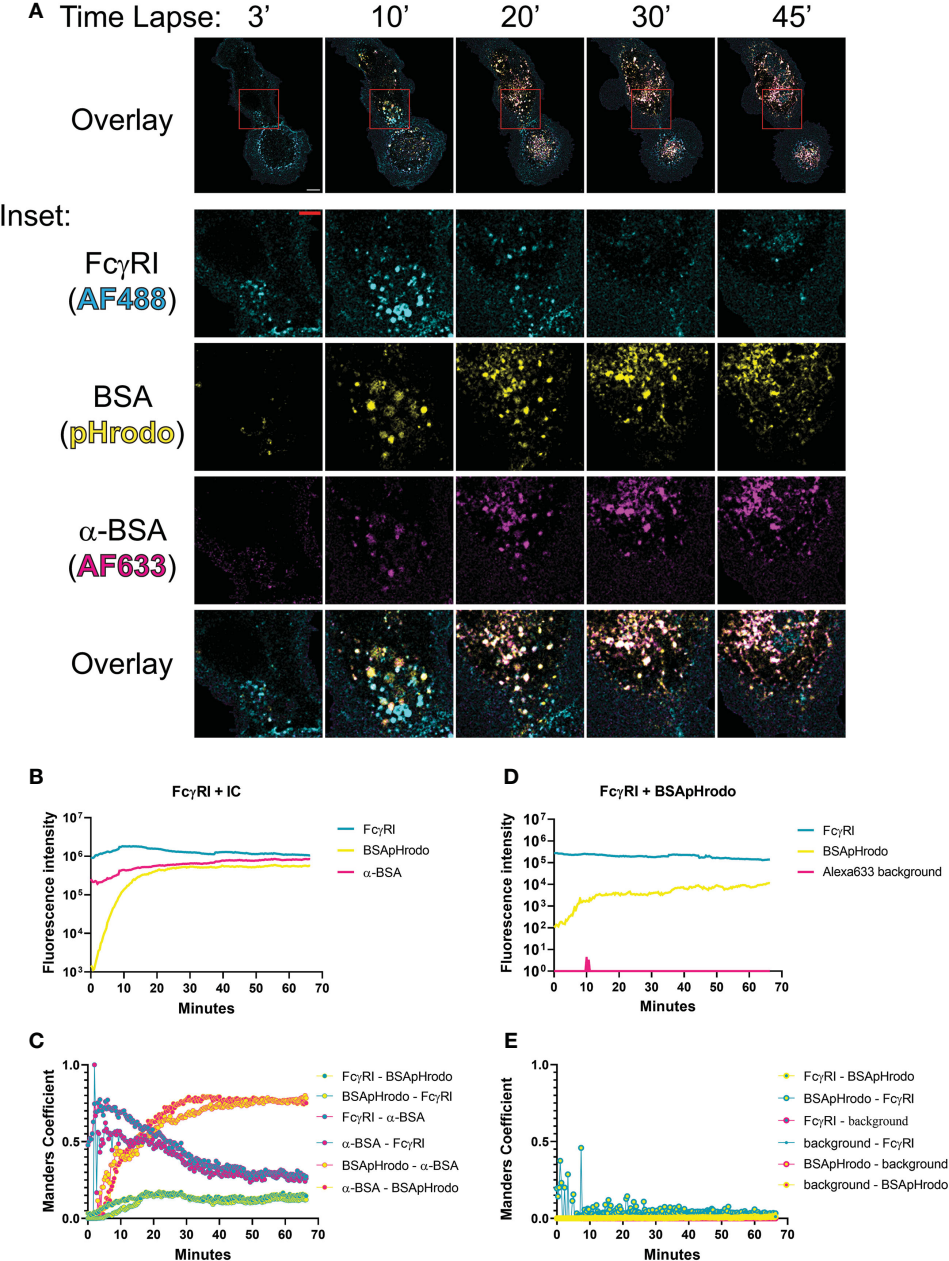
Temporal-spatial interaction between FcγRI and IgG during internalization of immune complex (IC). **(A)** Time lapse of fluorescent images showing FcγRI-mediated phagocytosis of IC by live monocyte-derived macrophages. The boxed area (10μm x 10 μm) was further enlarged to show the distribution of IgG(Alexa633), BSA(pHrodo) and FcγRI(Alexa488) with a scale bar of 5μm. **(B, D)** Time-dependent fluorescent intensities of labeled FcγRI, BSA and anti-BSA during FcγRI-mediated internalization of BSA-anti-BSA immune complex **(B)** or its control without anti-BSA **(D)**. The fluorescence of pHrodo increased as the pH declined. More BSA are internalized in FcγRI-mediated process **(B)** than spontaneous internalization **(D)**. **(C–E)** Manders coefficient analyses of live cell images for colocalization between pairs of fluorescent probes during FcγRI-mediated internalization of BSA-anti-BSA immune complex **(C)** or its control without anti-BSA **(E)**. Manders coefficients were calculated for pairwise colocalization between FcγRI, BSApHrodo, and anti-BSA antibody with respect to either fluorescent probe. For example, FcγRI-α-BSA refers to the percentage of FcγRI (cyan) that colocalizes with anti-BSA (magenta), while α-BSA-FcγRI is magenta-cyan and is the percentage of α-BSA that colocalizes with FcγRI. While BSA and anti-BSA remain colocalized throughout the duration of the live cell imaging, FcγRI showed optimal colocalization with anti-BSA at the beginning and dissociated at a later time. FcγRI was stained by Alexa488 antibody(cyan), BSA protein was labeled with pHrodo(yellow), and anti-BSA antibody was labeled with Alexa633 (magenta).

To this point, we combined high resolution structures of H174R FcγRI variant/Fc complex with pH titration binding studies to reveal that previously published structures by Lu. et.al and Kiyoshi, et.al represented the high affinity and lower affinity binding of FcγRI to IgG, respectively. The binding of FcγRI to immune complexes leads to receptor endocytosis to late endosome and lysosome compartments for antigen presentation (9, 24, 25). This effector function of FcγRI is a continuous cellular process spanning from cell surface to intracellular organelles that undergoes pH changes from neutral pH to pH 4.5-5 inside lysosomes (26). To assess the temporal-spatial interaction between FcγRI and IgG during this biological process, we used bovine serum albumin (BSA) and polyclonal rabbit anti-BSA antibodies to form a model immune-complex and examined the Fc receptor-mediated internalization of BSA immune complex labeled with a pH sensitive fluorescence probe, pHrodo, in human monocyte derived macrophages by live cell fluorescence microscopy (**Figure 7** and **Movie S1**). As mentioned above, BSA/anti-BSA antibody interaction was stable between pH 7 and 4, whereas FcγRI and anti-BSA interaction was pH sensitive, similar to that between FcγRI and human IgG (**Figure 6**). The dependence on the Fc receptor in the internalization of the immune complex was evident as the internalization of BSA was minimal in the absence of anti-BSA antibody and FcγRI was distributed exclusively on the cell surface (**Figures 7D, E** and **Movie S2**). To quantify this dependence in cells, we carried out a colocalization analysis on time lapse images of live monocyte-derived macrophages, comparing the colocalization over time of FcγRI, IgG, and pHrodo using the Manders Colocalization Coefficient (MCC), which is the percentage of overlapped signal in the channel and ranges from 0 (no colocalization) to 1 (total colocalization) (**Figure 7C**) (27). Imaging started by addition of preformed pHrodo-BSA-anti-BSA-IgG complexes and FcγRI antibody to the cell media. In the first ~5-10 minutes, FcγRI and anti-BSA IgG were at peak overlap levels with little pHrodo fluorescence, indicating that FcγRI interacted with anti-BSA IgG at neutral pH. For the next ~10-15 minutes following this initial period, pHrodo fluorescence increased rapidly, indicating a drop in the intra-organelle pH (**Figure 7B**). During this transitional period, colocalization between anti-BSA and FcγRI was high but began to decline, indicating FcγRI-mediated internalization of immune complexes but increasing instability of the complex. Also, during this period, BSA/pHrodo demonstrated increasing overlap with anti-BSA IgG, indicating stable association of BSA-anti-BSA-IgG within increasingly acidic intracellular compartments. Following this transitional period, the overlap between FcγRI and anti-BSA declined, indicating a pH sensitive dissociation between FcγRI and IgG (**Figure 7C**). Thus, our timelapse imaging of FcγRI-mediated internalization of immune complexes in live cells demonstrates that the pH-dependent FcγRI-IgG interaction mediates FcγRI effector functions. The observed pH-sensitive interaction of the FcγRI FG-loop to Fc suggests that the high affinity receptor FG-loop may function as a switch for immune-complex release in the low pH lysosomal compartment.

### Antibody-antigen uptake and release by FcγRI

**Figure 8:**
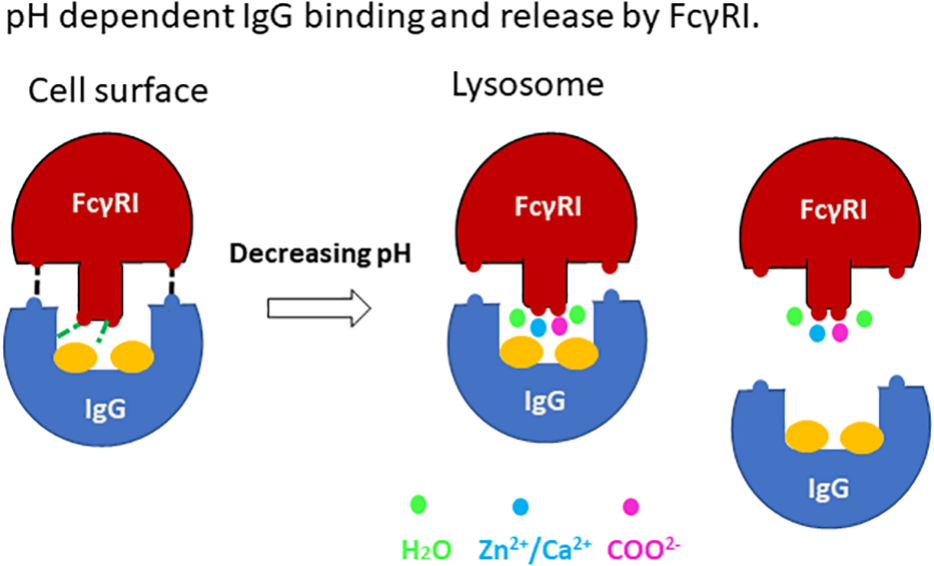
A cartoon showing the pH dependent IgG binding and release by FcγRI.

Our work presented here supports a structural mechanism for immune complex uptake and release by the high affinity FcγRI (**Figure 8**). The uptake of immune complexes on the cell surface involves binding of FcγRI to the lower hinge region of antibodies in a mode similar to the docking of antibodies to the low affinity Fcγ receptors. The binding of antibody enables FcγRI D2 domain FG-loop to engage Fc and its conserved glycan associated with Asn 297. These FG-loop and Fc interactions provide a high affinity receptor binding to immune-complexes. Upon internalization of the immune complex to lysosomal compartments, the low pH environment with increased concentrations of acetate promotes the receptor FG-loop binding to acetate and cations. Their synergistic effect would trigger the release of the FG-loop from Fc to allow FcγRI to adopt a low-affinity binding mode as observed by Kiyoshi et al. in the lysosomal compartments. The subsequent loss of high affinity IgG binding to allow immune-complex dissociation from the receptor.

The pH sensitive high affinity IgG binding is unique to FcγRI and enables the receptor to capture small antibody-antigen complexes for internalization and antigen presentation. Unlike FcγRI, the low affinity FcγRII and FcγRIII require binding avidity for their function and can only capture large, aggregated immune complexes for internalization and phagocytosis. The low affinity Fc receptors binding to smaller soluble oligomeric or monomeric immune complexes would result in early endosomal release rather than lysosomal delivery (**Figure 6D**). For example, cartilage damage in experimental antigen-induced arthritis (AIA) using methylated BSA (mBSA) was largely dependent on FcγRI but not FcγRIII (28).

## Discussion

The determination of several crystal structures of the high affinity human FcγRI-Fc complexes offered a rare opportunity for insight into the function of the receptor. All complexes showed similar receptor-Fc docking modes, with the D1 and D2 domains of FcγRI docking onto the lower hinge region of IgG-Fc in similar orientations as the respective D1 and D2 domains of all low affinity Fcγ receptors. The main differences among these structures reside in the conformation of the D2 domain FG-loop: residues 171-176 of FcγRI. The structural differences appear to be associated with their crystallization conditions. The complex structures presented here, as well as the previous one by Lu et al. (4X4M), were crystallized at neutral pH without acetate and both showed the FG-loop interacting with Fc and its glycans. While the receptor FG-loop in the current structures and 4X4M both formed a salt bridge with D265 of Fc-B and contacted the neighboring Fc glycan, the specific receptor-Fc pairing varied due to the H174R change. Arg 174 in the current structure forms both the salt bridge with D265 and the hydrogen-bond with the second GlcNAc on Fc-B. In the wildtype FcγRI complex (4X4M), the salt bridge to D265 was formed by Lys 173 of FcγRI while the glycan contact was mediated by His 174. In the structures by Kiyoshi et al. and Oganesyan et al., the corresponding FG-loop does not make direct contacts with Fc, and instead, is coordinated by Zn, acetate, and water molecules. Indeed, FcγRI binding to IgG_1_ is pH sensitive exhibiting ~10nM affinity at neutral pH when the FG-loop is engaged to Fc but reduced to ~200 nM affinity at pH 4.6 when the FG-loop is disconnected from Fc, suggesting the FcγRI FG-loop improves the receptor-IgG affinity ~20-fold from 200nM to 10nM. This is also consistent with the IgG binding affinity of the FG-loop AAA mutant at neutral pH. In contrast, both the FcγRI hydrophobic pocket, formed by Trp 104, Leu 105, Lys 130, Val 132 and Tyr 176, and its contacts with Leu 235 of Fc were nearly identical between complex structures solved at neutral and acidic pH (**Figures 4H, I**), suggesting the hydrophobic contacts between FcγRI and Leu 235 of Fc are not responsible for the pH dependent receptor-IgG binding.

To this end, the question of how FcγRI gained its 100-fold IgG binding affinity over the low affinity FcγRII and FcγRIII has been settled. Our structural and mutational analyses suggest the presence of three primary structural components contributing to the high affinity FcγRI binding to IgG. The most significant contribution to the high affinity FcγR is the interaction between the positively charged FcγRI D2-domain FG-loop and Fc as well as its associated glycan. These receptor FG-loop and Fc contacts contribute approximately 20-fold increase in IgG binding affinity over FcγRIII. Second, FcγRI forms better van der Waals contacts with Fc than FcγRIII. There are three interfaces with van der Waals clusters between FcγRI and Fc but only two between FcγRIII and Fc (**Tables S2, S3**). The additional cluster was unique to FcγRI and was proposed by Kiyoshi et al. as the primary source for FcγRI’s high affinity IgG binding. Mutation of the cluster resulted in ~2-fold reduction in IgG binding affinity (**Figure 5C**). Even among the common interfaces van der Waals clusters, FcγRI forms better hydrophobic packings than FcγRIII (**Table S2**), suggesting they contribute to a more favorable IgG binding in FcγRI than FcγRII and FcγRIII. Third, FcγRI forms more hydrogen-bonds with Fc at their lower hinge contact area than the low affinity FcγRIII. There are 3-6 hydrogen-bonds at the lower hinge contacts between FcγRI and Fc, but only one observed between FcγRIII and Fc (**Tables S2, S3**).

The relevance of FcγRI in cellular function and immune response has been long debated. The fact that circulating IgG concentrations are three orders of magnitude higher than the receptor-IgG dissociation constant suggests most cell surface expressed FcγRI would be occupied by monomeric IgG, making them unavailable for pathogenic immune complexes (3, 5). Nevertheless, the FcγRI-deficient animals exhibited defects in phagocytosis of immune complexes, antigen presentations and antibody-mediated therapies (9, 29). We provided structural and biochemical evidence to show that the high affinity IgG binding of FcγRI is pH sensitive, likely suggesting a functional role of FcγRI is to internalize small monomeric antibody-antigen complexes for antigen presentation. Consistent with this view, we suggest that the distinct FG-loop contacts observed in different pH environment represent two distinct states of the receptor: the high affinity immune complex binding state of the receptor on cell surface and its low affinity antigen release state (30, 31).

Current work demonstrates an important role of IgG-Fc associated glycans in FcγRI function. Earlier work supports the function of Fc-glycans in maintaining the lower hinge conformation of Fc to be compatible for low affinity Fc receptor binding (32, 33). Other than the role in conformational stability, Fc glycans were not observed to contact receptors directly in low affinity Fc-receptors (18, 19). It’s worth noting that the FcγRI FG-loop-mediated high affinity IgG binding mechanism is distinct from that of FcϵRI binding to IgE, which does not involve the corresponding receptor FG-loop but contains more extensive interface contacts that are stabilized by IgE-Cϵ2 domain (34, 35). The involvement of Fc-glycan in the high affinity FcγRI binding to IgG suggests potential use of antibody glycan engineering to modulate its binding to FcγRI, and thereby achieve desired receptor binding affinity for optimum therapeutic applications.

## Methods

### Expression and purification of recombinant proteins

IgG1 heavy and light chain expression plasmids of anti-HIV gp120 antibody VRC01 were kindly provided by Dr. Peter Kwong(VRC/NIAID). VRC01 heavy chain D265R, FcγRI and FcγRIIIA mutations were generated by site-directed mutagenesis using a QuikChange II Site-Directed Mutagenesis Kit (Agilent) according to the manufacture’s instruction. All mutations were confirmed by DNA sequencing (ACGT Inc.). The mutated FcγRI proteins, H174R variant, KHR/EEE triple mutant, and V132L/Y176V double mutant were expressed as the wild type and purified by IgG-sepharose affinity or Ni-NTA chromatography as described previously (12). FcγRIIIA wild type protein, and L118V, V164Y, or L118V/V163Y mutants were expressed as inclusion bodies in bacteria and refolded as described previously (36). CHO DXB-11 cell-expressed Human IgG_1_-Fc protein (216–444) was provided by Zymogenetics (Bristol-Myers Squibb Inc). Human plasma IgG_1_, IgG_3_, and IgG_4_ antibodies are purchased from Athens Research & Technology. Wildtype and D265R mutant VRC01 were expressed in 293freestyle cells (Thermo Scientific) by transient transfection with polyethylenimine (Polysciences). The transfected cells were cultured in a shaker incubator at 120rpm, 37°C, 8% CO2 for 3-4 days. Culture supernatants were harvested and secreted antibodies were purified through a protein A column. All proteins were further purified by a Hiload 16/600 Superdex 200 column (GE healthcare).

### Crystallization and structure determination

Before crystallization, FcγRI H174R variant was mixed with Fc dimer at 1.2:1 molar ratio and the FcγRI –Fc complex was further purified by gel-filtration chromatography in 10mM Hepes (pH7.4) and 0.15M NaCl and concentrated to OD_280nm_ of 18. Crystals were grown directly using commercially available screens from Hampton research and Qiagen in sitting-drop crystallization experiments that were set up in 96-well InterlliPlates by a Phoenix crystallization robot (Art Robbins Instruments). FcγRI H174R variant-Fc complex crystals grew in conditions containing 20% PEG3350 with either 0.2M of sodium formate (pH6.7), magnesium formate (pH7.0), ammonium formate (pH6.6), sodium sulfate (pH6.7), potassium thiocyanate(pH7.0), sodium thiocyanate (pH6.9) or magnesium sulfate (pH6.0). The crystals were immersed in the above mother liquors plus 15% glycerol as the cryoprotectants prior to flash-cooling in liquid nitrogen. X-ray data sets for crystals grown in 20%PEG3350 and 0.2M sodium formate, or 0.2M magnesium formate, or 0.2M magnesium sulfate, were collected to 2.3 Å and 2.5 Å resolution respectively at SER-CAT beamlines, processed and merged with HKL2000 (37) (**Table S1**). The structure of the FcγRI H174R-Fc complex was solved by a molecular replacement method with the program Phaser (38) in CCP4 packages (39) using FcγRI (PDB ID: 3RJD) and Fc (PDB ID: 3AY4) as the search model, respectively. Model building and refinement were carried out using Coot (40) and Phenix (41). The overall electron density of the complex is of excellent quality and carbohydrate molecules were added manually using (2Fo-Fc) electron density maps contoured at 1.0σ (standard deviation of the map) and refined. The residues are numbered consistent with FCGR1_HUMAN in the Swiss-Prot entry. The Ramachandran statistics were generated and verified by Procheck of CCP4. The buried surface area was calculated and all structure figures were generated using Pymol (42). The final models of refined structures and X-ray diffraction data were deposited into Protein Data Bank with codes of 8DIR, 8DIN and 8DJ7, respectively

### Surface plasmon resonance and biolayer interferometry binding

Surface plasmon resonance measurements were performed using a BIAcore 3000 instrument and analyzed with BIAevaluation 4.1 software (Biacore AB). Different human IgG subclasses were obtained from Athens Research and Technology (Athens, GA). To measure the affinity to wild type or mutant FcγRI and FcγRIII proteins, human IgG_1_, IgG3 or IgG_4_ were immobilized on carboxylated dextran CM5 chips (Biacore AB) to 200-1000 response units (RU) using a primary amine-coupling in 10mM sodium acetate (pH 5.0). To measure the pH-sensitive binding between FcγRI and IgG1, FcγRI, H174R and H174E variants were immobilized on CM5 chips. The analytes consisted of serial dilutions of IgG1 between 2µM and 63 nM in a buffer containing 0.15M NaCl plus 10mM Hepes (pH7.4), or 10mM MES (pH6.5-5.8), or 10mM sodium acetate(pH5.6-4.0). The dissociation constants were obtained by kinetic curve fitting for the binding of FcγRI to IgGs, and steady-state fitting for the binding of FcγRI mutants to IgGs, respectively, using BIAevaluation 4.1 (BIAcore Inc.). For BLI binding experiments, VRC01 and D265 mutant antibodies were captured to Octet Protein A(ProA) biosensors using an Octet R8(Sartorius) to a response level of ~5nm. The sensortips were then dipped into running buffer of 10mM Hepes(pH7.5), 0.15M NaCl to remove unbound IgG. To measure association, the sensortips were dipped into wells containing serial dilutions of FcγRI (Zymogenetics) before being dipped into wells only containing running buffer to measure dissociation. Data were reference subtracted and fit into a 1:1 binding model using Octet data analysis software v12.2.

### Live cell imaging of FcγRI-mediated immune complex uptake and release

Monocytes from healthy volunteers were obtained from individuals who consented to and enrolled in the NIH clinical center blood bank protocol (ClinicalTrials.gov NCT00001846). Human monocyte-derived macrophages were generated by culturing monocytes in RPMI 1640 supplemented with 10% FBS, 10ng/ml M-CSF, 10ng/ml GM-CSF, 20mM L-glutamine and 50U/ml penicillin-Streptomycin for 7 days. After that, ~50,000 macrophages were plated in 8-well confocal cover slides for 1 h at 37°C before imaging. Polyclonal rabbit anti-BSA IgG and BSA were labeled with Alexa633 and pHrodo (Thermo Scientific) according to manufacturers’ instruction, respectively. To test the binding of anti-BSA IgG to human FcγRI, elutriated monocytes were stained with monomeric polyclonal rabbit IgG (Alexa633) at different concentrations ranging from 6-200ug/ml in the absence or presence of 5μg/ml either anti- FcγRI antibody (Biolegend) or its isotype control (Alex488). Flow cytometry was performed on Fortessa X-20 (BD Biosciences) and data were analyzed using FlowJo software (www.flowjo.com). The presence of anti**-** FcγRI antibody does not interfere the binding of anti-BSA IgG to human FcγRI (**Figure S2**). To form immune complex, 10ul labelled BSA(4mg/ml) was added dropwise to 50ul anti-BSA IgG(2mg/ml) in 1x PBS and incubated for 10min on ice. Right before imaging, 20ul immune complex and 2ul Alexa488 labelled CD64 antibody (Biolegend) (0.5mg/ml) were added to macrophages with 180ul imaging solution of 1xPBS plus 5% FBS. Imaging was performed with a Zeiss LSM 880 confocal microscope while maintaining incubation conditions at 37°C, 5% CO_2_, in a humidified chamber. Images were acquired at 20 s interval for 1 hr. Images were denoised and background subtracted (10 px radius) using ImageJ 1.53s. Extraneous signal outside the bounds of the differential interference contrast (DIC) cell outlines were removed. Manders Colocalization Coefficients (MCCs) were calculated using a custom macro and Chosen thresholds were guided by Costes regression analysis (43).

## Data availability statement

The datasets presented in this study can be found in online repositories. The names of the repository/repositories and accession number(s) can be found below: http://www.wwpdb.org/, 8DIR, 8DIN, 8DJ7.

## Author contributions

JL and PS contributed to conception and design of the study. JL, MS, JB, MT, ZZ performed the experiments, JL and PS wrote the first draft of the manuscript. MS wrote sections of the manuscript. All authors contributed to the article and approved the submitted version.
