# Supplementary material for: FcγRI FG-loop functions as a pH sensitive switch for IgG binding and release
Source: Front Immunol. 2023 Feb 6;14:1100499. doi: 10.3389/fimmu.2023.1100499 (PMC9940316; doi:10.3389/fimmu.2023.1100499)
Supplement: Supplementary file 1 [file DataSheet_1.docx]

**Figure S1**. A) and B) (2Fo-Fc) electron density map (blue:1σ and cyan: 0.8σ) showing FG loop of FcγRI, D265 and glycans on Fc in crystals from magnesium formate (PDB ID 8DIN) and magnesium sulfate(PDB ID 8DJ7), respectively.


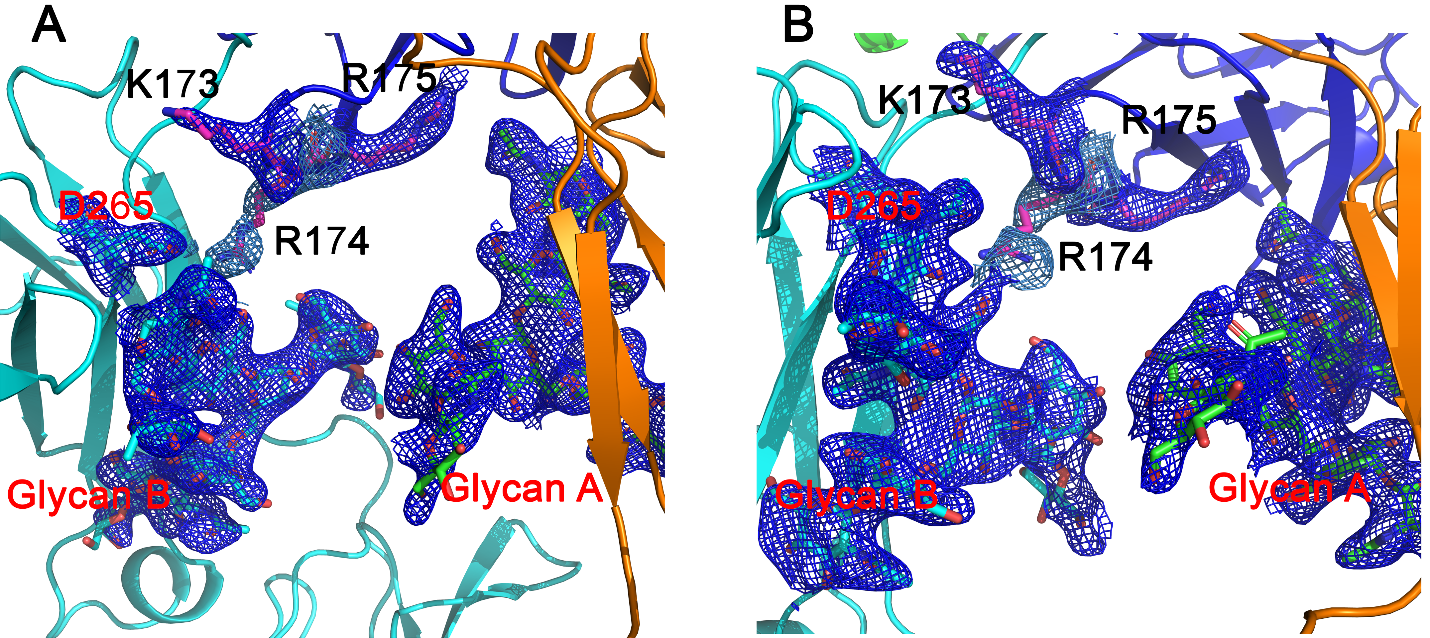


Figure S2. Co-staining of monocytes with anti- FcγRI antibody and monomeric rabbit polyclonal IgG. A)Representative flow cytometric analysis of monocytes stained with monomeric IgG (Alexa633), anti- FcγRI or isotype control(Alexa488) or in combinations. Data from three independent experiments were summarized in panel B) and C). B)Mean fluorescence intensity (MFI) of monomeric IgG (Alexa633) staining of monocytes in the absence and presence of 5μg/ml anti- FcγRI antibody or its isotype control(Alexa488). C)MFI of staining of monocytes with 5μg/ml anti- FcγRI antibody or its isotype control (Alexa488) in the absence or presence of various concentrations of monomeric IgG(Alexa633).


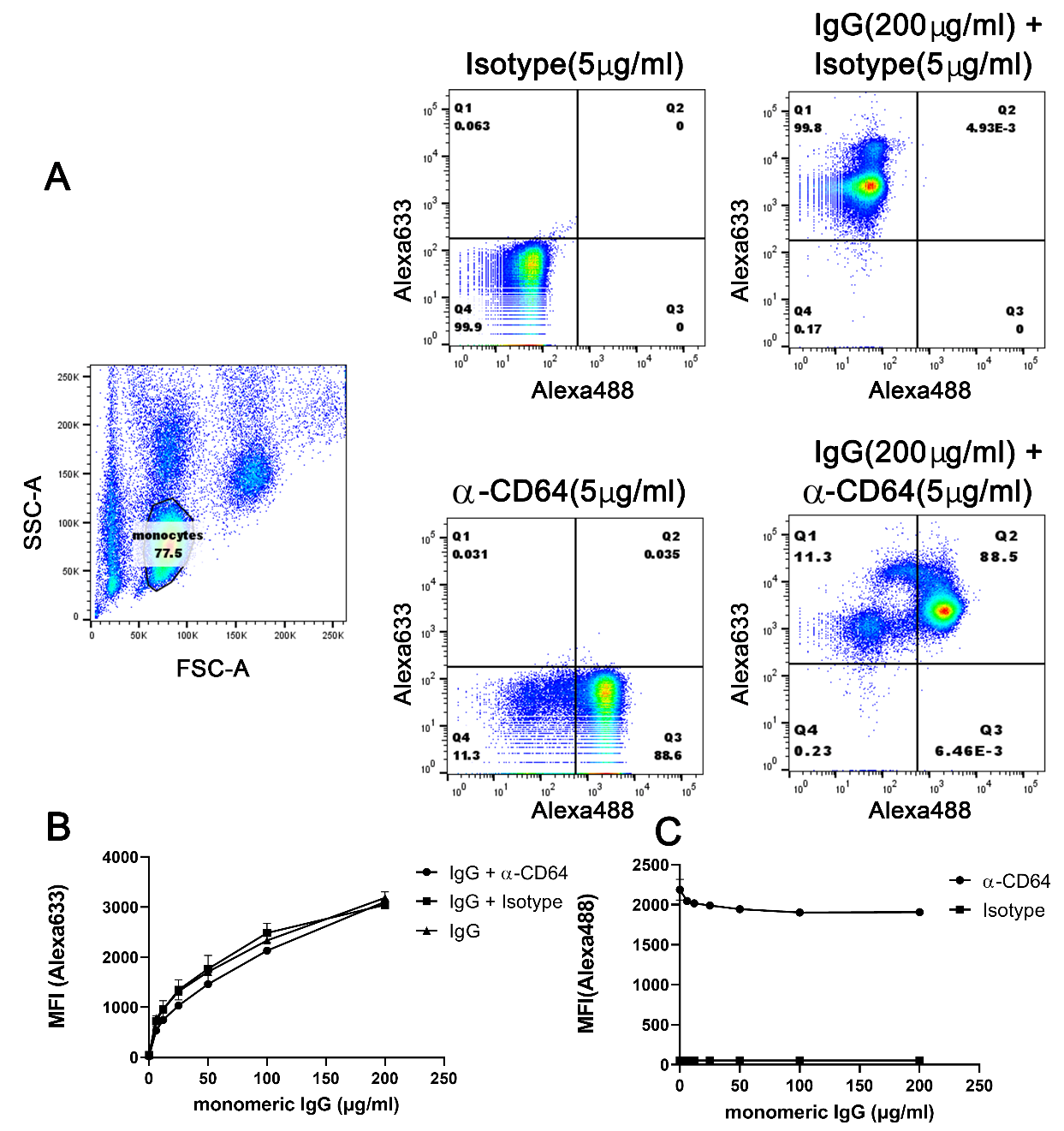


**Table S1**. Crystallographic parameters of three H174R FcγRI-Fc complex structures

| **Data collection** | | | |
| --- | --- | --- | --- |
| PDB entry code | 8DIR | 8DIN | 8DJ7 |
| Crystallization condition | 20% PEG3350, 0.2M  Sodium Formate(pH6.6) | 20% PEG3350, 0.2M  Magnesium Formate | 20% PEG3350, 0.2M  Magnesium Sulfate |
| Space group | C2 | C2 | C2 |
| Unit cell dimension (Å) | a=204.3,b=89.3,c=56.1 b=97.7 | a=204.0,b=88.8,c=55.9 b =98.1 | a=206.4,b=89.01,c=56.2 b =96.8 |
| Resolution range (Å) * | 50-2.30(2.34-2.30) | 50-2.48(2.53-2.48) | 50-2.49(2.52-2.49) |
| Unique reflections | 44290(2235) | 34744(2137) | 34006(1948) |
| Average redundancy | 6.5(6.1) | 5.3(3.8) | 5.6(6.4) |
| Rmerge(%)a | 7.0(46.4) | 11.8(57.9) | 13.1(57.8) |
| I/σ(I) | 25.3(2.6) | 14.1(1.5) | 15.3(2.90) |
| Completeness (%) | 99.8(99.9) | 98.2(97.8) | 96.1(99.9) |
| **Refinement statistics** | | | |
| Refinement resolution (Å) * | 36.87-2.3(2.35-2.30) | 36.77-2.50(2.57-2.50) | 37.17-2.39(2.46-2.39) |
| Rcryst (%)b | 19.57(30.64) | 19.08(31.22) | 21.41(34.06) |
| R_free_ (%)^c^ | 23.32(34.83) | 22.89(39.35) | 26.55(36.04) |
| Protein atoms | 5493 | 5493 | 5493 |
| ligands | 2GAL,8NAG,2BMA,2FUC,  6MAN,244 H2O | 2GAL,8NAG,2BMA,2FUC,  6MAN,127 H2O | 2GAL,8NAG,2BMA,2FUC,  6MAN,122 H2O |
| R.m.s deviation from ideal values | | | |
| Bond length (Å) | 0.009 | 0.009 | 0.004 |
| Bond angle (°) | 0.78 | 1.593 | 0.728 |
| Mean B-factor (Å^2^) | 63.41 | 66.81 | 69.66 |
| Wilson plot B-factor (Å^2^) | 43.45 | 50.44 | 51.03 |
| Ramachandran statistics | | | |
| Most favored region (%) | 97.96 | 96.5 | 96.3 |
| Additionally allowed (%) | 2.04 | 3.5 | 3.7 |
| a R_merge_ = ∑_h_∑_i_⎜I_i_(hkl) - < I(hkl)>⎜/ ∑_h_∑_i_I_i_(hkl), ^b^ R_cryst_ = ∑⎜⎜Fo⎟ - ⎟Fc⎟⎟ / ∑⎟Fo⎟ calculated from working data set. ^c^ Rfree is  calculated from ~5% of data randomly chosen not to be included in refinement. *Values for the highest resolution shell were listed in the parenthesis. | | | |

**Table S2.** FcR- Fc interface contacts in H174R, 4W4O and 1T83.

|  | **FcγRI-Fc complex** | | | | | | **FcγRIII-Fc complex** | | |
| --- | --- | --- | --- | --- | --- | --- | --- | --- | --- |
|  | Lu et al (H174R) | | | Kiyoshi et al. (4W4O) | | | Radaev et al (1T83) | | |
| **Hinge-A** | **FcγRI** | **Fc** | **Type** | **FcγRI** | **Fc** | **Type** | **FcγRIII** | **Fc** | **Type** |
| Salt Bridge | K142 | Y296 | HB | K142 | Y296 | HB |  |  |  |
|  | K145 | E269 | SB | K145 | E269 | w- HB | K131 | E269 | SB |
| H-bond | N134 | G237 | HB | N134 | G237 | HB | K120 | D265 | SB |
|  | K145 | S298 | HB |  |  |  |  |  |  |
|  | F146 | H268 | w-HB | F146 | H268 | w-HB |  |  |  |
|  | H148 | D265 | HB | H148 | D270 | SB | H134 | G237 | HB |
|  | A143 | Y296 | w-HB | A143 | Y296 | w-HB |  |  |  |
| Van der Waals | Y133 W149 | L235 G236 | VDW | Y133 W149 | L235 G236 | VDW | H119 H135 |  | VDW |
|  | | | | | | | | | |
| **Hinge-B** | **FcγRI** | **Fc** | **Type** | **FcγRI** | **Fc** | **Type** |  |  |  |
| H-bond | R102 | P329 | HB | R102 | P329 | HB |  |  |  |
| VDW | W104 W127 | L328 P329 | VDW | W104 W127 | L328 P329 | VDW | W90 W113 |  | VDW |
|  | W104 L105 K130 V132 Y176 | L235 | VDW | W104 L105 K130 V132 Y176 | L235 | VDW | A117 V158 G159 | L235 | VDW |
|  | | | | | | | | | |
| **FG-loop** | **FcγRI** | **Fc** | **Type** | **FcγRI** | **Fc** | **Type** |  |  |  |
|  | R174 | NAG | HB |  | | |  |  |  |
|  | R174 | D265 | SB | H174 | D265 | w-HB |  |  |  |
| SB: salt bridge; HB: hydrogen bond, w-HB: water-mediated hydrogen bond; VDW: Van der Waals interaction. | | | | | | | | | |

**Table S3**. FcγRI-Fc interface contacts

| **Total receptor-Fc contacts** | **H174R** | **4W4O** | **FcγRIII-Fc** |
| --- | --- | --- | --- |
| Salt bridges | 2 | 1 | 2 |
| H-bonds | 6 | 3 | 1 |
| Water-mediated H-bonds (w-HB) | 2 | 3 |  |
| VDW clusters | 3 | 3 | 2 |
| Bridging waters | 4 | >16 |  |

**Movie S1**. Live cell imaging of FcγRI mediated internalization of immune complex by macrophages. FcγRI was stained by Alexa488 antibody(cyan)(upper left panel), BSA protein was labeled with pHrodo(yellow)(upper right panel), and anti-BSA antibody was labeled with Alexa633 (magenta)(lower left panel). The lower right panel shows the merge of all three channels.

**Movie S2**. FcγRI could not mediate antigen uptake by macrophages in the absence of antibody. FcγRI was stained by Alexa488 antibody(cyan)(upper left panel), and BSA protein was labeled with pHrodo(yellow)(upper right panel). The lower right panel shows the merge of the two channels.
